# A Critical Comparison of Focused Ethnography and Interpretive Phenomenology in Nursing Research

**DOI:** 10.1177/23333936241238097

**Published:** 2024-03-14

**Authors:** Uchechi Clara Opara, Pammla Petrucka

**Affiliations:** 1 University of Saskatchewan, Saskatoon, Canada

**Keywords:** interpretive phenomenology, focused ethnography, nursing, research

## Abstract

Choosing an appropriate qualitative methodology in nursing research is a researcher’s first step before beginning a study. Such a step is critical as the selected qualitative methodology should be congruent with the research questions, study assumptions, data gathering and analysis to promote the utility of such research in enhancing nursing knowledge. In this paper, we compare focused ethnography by Roper and Shapira and interpretive phenomenology by Benner. Though these methodologies are naturalistic and appear similar, both have different methodological underpinnings. The historical, ontological, epistemological, and axiological philosophy guiding each methodology are described. In addition, the methodological underpinnings of both methodologies and a justification for use in nursing research are provided. This paper will assist future researchers who aim to employ these methodologies in nursing research.

Qualitative methodologies are research approaches that explore, narrate and explain human experiences and phenomena to promote clarification, interpretation, and understanding of such phenomena within the context of the experiences (Curtis & Keeler, 2022a; Dyar, 2022; Renjith et al., 2021). Qualitative methodologies have been used extensively in nursing and allied disciplines to explain individuals’ experiences, understand human behavioral patterns, explore healthcare needs, develop theories, and design health interventions (Curtis & Keeler, 2022a; Dyar, 2022; Ghafourifard et al., 2022; Kim & Chang, 2022; Polit & Beck, 2021). The choice of a particular qualitative research methodology is dependent on the research question and a critical analysis of how such methodology is a fit and able to provide in-depth exploration, understanding, and interpretation of a research problem (Busetto et al., 2020; Dyar, 2022; Polit & Beck, 2021). Such a decision must be documented and discussed when reporting the methods and results section of qualitative research (Busetto et al., 2020). Appropriate use of qualitative research methodologies in nursing could promote an in-depth understanding of people’s experiences to enhance interventions, health outcomes, nursing education, and practice (Busetto et al., 2020; Dyar, 2022; Kim & Chang, 2022; Polit & Beck, 2021; Ryan, 2017). However, choosing a suitable qualitative methodology appropriate for specific nursing research could be challenging for novice nurse researchers who may lack an in-depth background and understanding of applying relevant methodological and philosophical underpinnings of diverse qualitative methodologies in research.

There are diverse qualitative methodologies used in nursing, such as phenomenology, ethnography, narrative, grounded theory, case study, and historical methodologies (Curtis & Keeler, 2022b; Dyar, 2022). Generally, focused ethnography (FE) is an evolving pragmatic and valuable methodology adapted from the traditional ethnographic methodology used to explore and understand distinct phenomena in a specific setting **(**Higginbottom et al., 2013; Wall, 2015 **)**. Additionally, interpretive phenomenology (IP) is a popular philosophical and methodological approach that allows for a unique capturing and revealing of the meanings and interpretations of phenomena based on intersubjective views of participants and researchers (Frechette et al., 2020). Both FE and IP have been developed across various academic disciplines and fields of knowledge ranging from sociology, education, nursing, and other related health disciplines such as public health, medical education and global health (Andreassen et al., 2020; Trundle & Phillips, 2023; Van Manen, 2014). However, in this paper, we will critically compare FE methodology described by Roper and Shapira (2000) with Benner’s IP inquiry based on Heidegger’s (1927/2019) phenomenological philosophy.

FE by Roper and Shapira (2000) is a contemporary anthropological approach that explores, narrates, and interprets collective cultural experiences within a limited timeline when compared to traditional ethnography (Cruz & Higginbottom, 2013; Roper & Shapira, 2000; Wall, 2015). FE described by Roper and Shapira (2000) in nursing aims to understand a specific situation or phenomenon within a subculture of a population to understand the meaning that specific members of a subculture assign to their experiences (Cruz & Higginbottom, 2013; Higginbottom et al., 2013; Roper & Shapira, 2000). On the other hand, Benner’s (1994) IP methodology, based on Heidegger’s (1927/2019) phenomenological philosophy, has been employed by nurses globally to explore, access, and understand meanings of taken-for-granted issues or phenomena in everyday life experiences. There are diverse types of FE and IP; however, our discussion will focus on FE described by Roper and Shapira (2000) and Benner’s (1994) IP methodology based on Heidegger’s phenomenological philosophy.

The rationale behind comparing FE, described by Roper and Shapira (2000), and IP, described by Benner (1994), is that though both methodologies differ, some aspects of their philosophical and methodological underpinnings are similar. For example, both methodologies are grounded on culture. Benner emphasizes that the background meanings of a phenomenon held by a person are enmeshed in the culture, the subculture and the family of the individual (Benner, 1994; Benner & Wrubel, 1989). Such ontological views of Benner (1994) are also consistent with Roper and Shapira (2000), who emphasize the need to explore a subculture by learning about people and from such people. These shared ontological perspectives also influence some similarities in the epistemology and methodological approaches in interviews and participant observations employed by these nurse researchers to ensure a depth of understanding of the phenomenon of interest. While these two methodologies may have some similar approaches to data gathering, there are differences in their goals, research questions and techniques employed to answer such questions, often making it challenging for students and novice researchers to decide which one to select for their research work, especially at this time that FE described by Roper and Shapira (2000) is evolving in nursing. Several nurse researchers, such as Munhall (1994; 2012) and Conroy (2003), have developed methodological approaches drawn from Heideggerian IP philosophy, thus contributing immensely to nursing knowledge development. However, as explained above, Benner’s (1994) IP ontological perspective stands more consistent with Roper and Shapira’s (2000) FE. Moreover, to our knowledge, no article has previously compared Roper and Shapira’s (2000) FE and Benner’s (1994) IP methodology.

Therefore, this paper is expected to clarify these similarities and differences to allow researchers to make appropriate decisions in selecting the methodology that best fits their research question and purpose. In this paper, we will provide a brief general history of FE and IP to promote an in-depth understanding of both methodologies. Additionally, the ontology, epistemology, axiology, and methodological underpinnings of FE described by Roper and Shapira (2000) and IP described by Benner (1994) will also be compared to enhance understanding of their similarities and differences.

## Historical Foundation of Ethnography

The word ethnography is a term adopted from the ancient Greek words “ethnos” (people and tribe) and “graphia” (writing) (Jones, 2010, p. 13). Ethnography emerged in Western civilization at the end of the 19th century and the beginning of the 20th century (Brewer, 2000). At that time, ethnography focused on the formal description of non-Western cultures, with researchers primarily employing the classic and armchair ethnographic methodologies (Hammersley & Atkinson, 2019; Ryan, 2017; Van Maanen, 1995 **).** Classical ethnographers based their data on fieldwork in remote non-Western cultures, while the armchair ethnographers relied on other researchers’ written accounts (Mayan, 2009; Medeiros & Cowall, 2020; Richards & Morse, 2013). However, both forms of ethnography took an objective and post-positivist stance, where researchers explore participants’ subjective experiences with limited integration of the researchers’ views and experiences (Fetterman, 2019). Such an objective stance in research limited an in-depth interpretation of the insider’s (emic) viewpoint and fostered ethnocentrism, where researchers viewed other cultures as primitive compared to Western culture (Medeiros & Cowall, 2020; Ryan, 2017; Smith, 2012).

A significant development in ethnography occurred in Europe between 1914 and 1922 (Nelson, 2020) when renowned anthropologists such as Bronislaw Malinowski redefined fieldwork and introduced active participant observation (Nelson, 2020; Rees & Gatenby, 2014; Ryan, 2017). Active participant observation allowed researchers to identify with the native’s way of life, which reduced ethnocentrism (Nelson, 2020). However, a researcher’s position as an active participant observer does not necessarily imply that such a researcher will gain a deep understanding of the participant’s perspective. Several factors, such as issues of trust, acceptance of the researcher in the context, and the researcher’s observation skills, play a significant role in the depth of participants’ views gained during observation (Holloway & Galvin, 2016; Kawulich, 2005).

Between the 1920s and 1950s, ethnographic research spread to the Chicago School of Ethnography in the United States, advancing ethnographic research study of the modern urban context (Hammersley & Atkinson, 2019; Ryan, 2017). In this context, there was a shift from researching exotic contexts of non-Western cultures to researching urbanization and industrialization of Western towns and villages (Hammersley & Atkinson, 2019; Nelson, 2020; Rees & Gatenby, 2014; Ryan, 2017). The ontological focus of research at this time was on cultural relativism, as researchers believed that culture cannot be objectively studied since humans see the world through the lens of their culture (Medeiros & Cowall, 2020; Nelson, 2020). Cultural relativism emphasizes understanding culture on its terms rather than being compared to an outsider’s standard (Medeiros & Cowall, 2020; Nelson, 2020). The focus on cultural relativism was a significant turning point in ethnographic fieldwork and distinguished research in the United States from European cultural anthropological research that maintained an objective stance in research at that time (Medeiros & Cowall, 2020).

By the 1960s, ethnography had spread globally and from sociology to other disciplines, making ethnography a multidisciplinary methodology (Hammersley & Atkinson, 2019; Rees & Gatenby, 2014). During this period (1960s), Leininger (2006) developed her ethno-nursing research methodology in response to the lack of a suitable ethnographic nursing research methodology to appropriately explore and promote an understanding of the nature and patterns of human care specific to nursing.

Geertz (1973), the founder of traditional postmodern ethnography, emphasized the importance of cultural immersion, where traditional postmodern ethnographic researchers engage in extensive fieldwork lasting over a year by living among a population to learn the culture’s language while participating in daily activities. Geertz’s (1973) notion of ethnography is based on the interpretive paradigm, where researchers can capture an in-depth understanding of the insider’s or emic perspective through a comprehensive account of the context, relationships, phenomena of interest, and the population to provide a thick description (Geertz, 1973). Using experience and knowledge, the researcher can create knowledge and draw conclusions on a phenomenon by interpreting the meaning attached to participants’ stories, intricate cultural symbols, and human interactions (Geertz, 1973).

### The Emergence of FE Methodology in Nursing

FE first emerged in nursing research in North America (Trundle & Phillips, 2023), with Morse (1991), a renowned qualitative nursing researcher, proposing FE as a distinct methodology. Morse’s (1991) FE proposal was further expanded by Muecke (1994), Boyle (1994), and Roper and Shapira (2000), further providing an extensive description of FE methodology. Given their knowledge of ethnographic research and training in anthropology, these researchers were able to carve out a pragmatic methodology that aligned with nursing research conducted within limited timelines. Other nursing researchers in the 2010s have further developed more detailed established boundaries that explicitly differentiate FE from traditional or classic ethnography while leaning heavily on Roper and Shapira’s (2000) FE account and data analysis approach. (Cruz & Higginbottom, 2013; Higginbottom et al., 2013; Rashid et al., 2019; Wall, 2015).

According to Roper and Shapira (2000), FE in nursing focuses on narrow research questions to study a distinct phenomenon within a population’s subculture in a limited timeline (Cruz & Higginbottom, 2013; Higginbottom et al., 2013; Roper & Shapira, 2000). Roper and Shapira’s (2000) FE methodology in nursing research aims to study nursing as a cultural phenomenon and explore how diverse cultures incorporate health beliefs and practices within their context (Cruz & Higginbottom, 2013; Higginbottom et al., 2013; Roper & Shapira, 2000). Research findings using this methodology seek to promote the integration of the cultural norms of members of a sub-cultures in nursing care to enhance health outcomes (Roper & Shapira, 2000). Though Roper and Shapira’s (2000) FE rapidly gathers context-focused and culturally relevant data within a limited time, it also intends to promote a thick description like traditional postmodern ethnography.

FE generally has been criticized due to the short immersion and narrow focus compared to traditional postmodern ethnography (Muecke, 1994). However, ethnography is defined by the depth of cultural description and not the volume of data (Roper & Shapira, 2000). Thus, integrating both the emic perspective (participants’ view) and the etic perspective (researchers’ view) in Roper and Shapira’s (2000) FE enhances the thickness of the data. Moreover, researchers’ theoretical knowledge of the phenomenon of interest and the population, mostly lacking in traditional postmodern ethnography, enhances ease of access to the population of interest and data interpretation (Andreassen et al., 2020; Higginbottom et al., 2013; Wall, 2015).

## Historical Foundations of Phenomenology

The phenomenological philosophy was first conceived by Husserl, a German mathematician and philosopher, in the 20th century (Annells, 1996; Dowling, 2007). Husserl objected to the philosophical views at a time when researchers embraced positivism while attributing limited value to human experience in knowledge development (Carman, 2006; Dowling, 2007; Munhall, 2012; Racher & Robinson, 2003). Husserl focused on understanding what can be known about the world (Dowling, 2007). Husserl emphasized that human experience is the basis for understanding, which could be objectively obtained by bracketing one’s preconceived views and ideas of a phenomenon (Husserl, 1913/2012; Koch, 1995). Husserl’s conception of phenomenology is situated in the post-positivist paradigm, as his notion of phenomenology is subjectively situated but allows for the objective exploration of an individual’s everyday life (Lincoln et al., 2018; Moran, 2000; Urcia, 2021). For Husserl, an individual’s experience of a phenomenon is characterized by features or “universal essence” apparent to the individual, which could influence a generalized description of that phenomenon (Dowling, 2007; Koch, 1995; Neubauer et al., 2019, p. 93).

However, Heidegger, a philosopher and a student of Husserl, also known as the father of hermeneutic phenomenological philosophy, posited that obtaining an unbiased account of human experience is impossible as humans cannot be separated from their political, historical, cultural, and social lifeworld (Annells, 1996; Burns & Peacock, 2019; Dodgson, 2023; Koch, 1995). In addition, Heidegger’s conception of phenomenology is situated in interpretivism; as such, Heidegger believes one’s preconceptions, knowledge, and views cannot be kept in abeyance or bracketed, as such promotes a detailed interpretation of the essence of a phenomenon (Amos, 2016; Cerbone, 2009; Koch, 1995; Mackey, 2005). Although Husserl and Heidegger acknowledged phenomenology as a vital philosophical approach that fosters understanding of human experience, they disagreed on the philosophical stance. For example, while Husserl’s phenomenology is epistemologically focused on one’s view of a phenomenon, Heidegger aimed to understand the knowledge that surpassed human experience, centering on the ontological view of the nature of being and time (Annells, 1996; Koch, 1995; Laverty, 2003). Thus, these variations in philosophical beliefs led to the development of Heideggerian IP.

Heidegger has been criticized for using his philosophical ideology to promote his political ambition, as he was active in the national socialist movement during the Second World War (Mackey, 2005). There are controversies about how his political ideology influenced his philosophical stance (Holmes, 1996). However, such criticism has not limited the use of Heidegger’s work in nursing research, suggesting that nurses are either “unaware or unmoved” by such criticism (Holmes, 1996, p. 579).

## Heideggerian Phenomenology in Nursing Research

Apart from Benner (1994), several nurse phenomenologists, such as Munhall (1994) and Conroy (2003), also embraced Heidegger’s phenomenological philosophy as a foundation to develop rich IP methodologies that address nursing issues. For example, Munhall’s (1994) IP allows a researcher to explore and interpret the meaning of a phenomenon experienced by individuals in the context of such experiences. Consequently, Munhall (1994) emphasizes the need for researchers to understand the significance of the historical context since interpretations of experiences have a cultural and linguistic connotation. In addition, Conroy’s (2003) IP focuses on interpreting the shifts and variations in people’s experiences of being in the world. These IP methodologies go beyond mere description to interpretation of complex phenomena such as pain, trauma, hope, and experiences of living with an illness (Conroy, 2003; Creswell & Poth, 2018; Munhall, 1994; 2012). While these phenomenologists (Benner, 1994; Conroy, 2003; Munhall, 1994) have varied aims and methodological underpinnings, they draw understanding and interpretations of human experiences from the ontological tenets of Heidegger’s phenomenological philosophy. However, these tenets will be discussed based on their application to Benner’s (1994) IP methodology, being the focus of this paper.

Heidegger believed that individuals are inseparable from their lifeworld, which he theorized in his use of the term, being in the world (Cerbone, 2009; Dreyfus & Wrathall, 2007; Heidegger, 1927/2019; Moran, 2000). Heidegger believed that individuals could exist in the world in many ways; one of the most significant ways of being in the world is understanding one’s existence, conceptualized as Dasein (Heidegger, 1927/2019). Heidegger (1927/2019) considers being in the world a priori aspect of Dasein. An individual’s existence, as Dasein, allows for a genuine understanding of one’s being (Gelven, 1989).

Heidegger also suggested that the fundamental principle of human understanding lies in our ability to interpret our everyday lives (Heidegger, 1927/2019). Such interpretation is considered by Heidegger as a hermeneutic process, which aims to unveil the understanding and interpretation of the meaning of an experience of being in the world through intersubjective perspectives (Benner, 1994). For Heidegger, interpretation reveals what has already been understood to unveil the meaning embedded in a phenomenon (Heidegger, 1927/2019). According to Lopez and Willis (2004), the hermeneutic process allows for an in-depth understanding of meanings that may not be obvious to an individual but are embedded in the person’s lifeworld. Therefore, for a researcher to co-create a holistic interpretation of an individual’s experience, the researcher must be grounded in the ontological tenets of IP, which emphasizes the need to understand the meaning of “Being” (Mackey, 2005, p. 181) instead of what can be known (Koch, 1995). Approaching the study from a relativist ontology, the researcher must also link the concept of being to the philosophical tenets of IP, embodiment, spatiality, temporality, care, and the hermeneutic circle to promote a deep awareness of human existence (Taylor, 2006). These tenets are discussed herein.

Benner defined embodiment as “skillful comportment and perceptual and emotional responses” (Benner, 1994, p. 104). Consequently, the body is viewed as a space through which an individual’s emotional experiences are expressed (Benner, 1994; Dos Santos et al., 2016). An individual’s body and emotional experiences allow a deeper understanding of experiences (Sharma et al., 2009). Understanding an individual’s embodied experiences is vital for a researcher to promote an in-depth interpretation of an individual’s experiences of a phenomenon.

Spatiality is also a feature of being in the world where individuals situated within space hold issues of interest closer while holding back other issues, which should not be confused with geographical space (Mackey, 2005). Conceptually, spatiality allows the researcher to listen to a participant’s experience and understand experiences that participants hold close to and those kept in abeyance (Mackey, 2005). However, most significant experiences are not always situated or recognized by participants as important experiences embedded in one’s being in the world (Burns & Peacock, 2019). Therefore, the researcher’s interviewing and probing skills could promote a deep exploration of participants’ experiences of a phenomenon. Such skills could also allow the researcher to understand experiences that a participant considers important and experiences that could be overlooked but are significant in the participant’s lifeworld (Mackey, 2005).

Heidegger posited that time is fundamental in understanding and interpreting all human experience and that temporality is the basis for our knowledge of our existence (Heidegger, 1927/2019). According to Heidegger (1927/2019), temporality allows for unity in an individual’s past, present, and future experiences. Therefore, an individual’s present experiences align with past experiences and possible future experiences (Orbanic, 1999). IP researchers must be aware of the concept of temporality and situate self, the participants, and their experiences in time in the interpretive process and evident in the description of the phenomena (Mackey, 2005). Such a process of interpretation promotes an understanding of how time influences an individual’s experience (Mackey, 2005).

For Heidegger, the words sorge and concern are interchangeable with care and are an essential component of Dasein or being in the world (Heidegger, 1927/2019). Heidegger (1927/2019) believes that through the act of caring, one can have a deeply meaningful experience of others, self, and the world. Additionally, care is relational with others and linked to time and context, as such care could be temporary or sustained over a long time (Heidegger, 1927/2019). Consequently, a researcher’s relational experience with participants could enhance the authenticity and understanding of participants’ experiences of a phenomenon (Burns & Peacock, 2019).

In explaining his notion of the hermeneutic circle, Heidegger stated that there is no circular reasoning but rather “relatedness backward or forward” between “being” and the phenomenon being explored (Heidegger, 1927/2019, p. 28; Tembo et al., 2023). Heidegger believes that the fore-structures of knowledge allow a back-and-forth movement between a partial to a more complete (whole) understanding (Heidegger, 1927/2019; Mackey, 2005). The fore-structure represents prior knowledge, or the background-based knowledge, perception, and experiences brought in by both the researcher and the participant (Benner, 1994; Mackey, 2005). Thus, using the hermeneutic circle, the researcher’s understanding of a text or a participant as a whole through reading and re-reading of a transcript is dependent on understanding a part of that text and how each part unfolds as a whole text as well as how the meaning and preconception in a text relates to other text or participants (Burns & Peacock, 2019; Ray & Locsin, 2023). However, Conroy (2003) emphasized that interpretation should follow a hermeneutic spiral instead of a closed circular understanding where the interpretation of meaning ends with the participants and the researcher. Conroy (2003) believes that the interpretation of meaning should be expanded to include other readers, which then releases the circle into a dynamic spiral that enriches the interpretation of meaning over time.

Based on the ontological tenets of Heidegger’s (1927/2019) interpretive philosophy, Benner (1994), a nurse researcher, developed an IP methodology to explore critical issues in nursing practice to provide a clear interpretation of “commonalities across participants' practical everyday understandings and knowledge” (p.103). In recognition of Heidegger’s (1927/2019) belief on interpretation, which seeks to reveal the meaning of being as hermeneutic or interpretive rather than a descriptive process, the researcher in Benner (1994) IP goes beyond mere description of the words of the participants to interpretation of the data provided by the participant. The researcher’s interpretation of the data begins when the researcher engages with the phenomenon of interest while listening to the participants’ descriptions of their experiences (Benner, 1994). The interpretive process is continuous as the researcher immerses self in the data while reading participants’ experiences of a phenomenon. Benner (1994) further stressed that to understand a phenomenon, the researcher should look beyond the phenomenon to the context in which the phenomenon was experienced to allow the researcher to better understand oneself, individuals, and the world. Additionally, the background knowledge and experience of both the researcher and the participant enhance understanding of the phenomenon of interest (Benner, 1994). Consequently, a researcher’s reflexivity is crucial to ensure the researcher acknowledges personal bias that could influence the research process. According to Benner (1994), an in-depth understanding of a phenomenon could enhance a more efficient, competent, and compassionate engagement in the nursing practice.

## Ontology of FE by Roper and Shapira (2000) and Benner’s (1994) IP

The ontological assumption or the nature of reality in Roper and Shapira’s (2000) FE and Benner’s (1994) IP are similar as both embrace a relativist or constructivist ontological view (Burns et al., 2022; Higginbottom et al., 2013; Ryan, 2017). Relativist ontology is of the view that multiple realities allow for a comprehensive understanding of multiple truths related to a phenomenon (Denzin & Lincoln, 2018; Ryan, 2017). Additionally, an individual’s experience is socially constructed and dependent on time, context, and social interaction (Denzin, 1989; Ryan, 2017). In FE by Roper and Shapira (2000), an individual is inseparable from the collective cultural experiences within a group based on their communication with others and the cultural norms, beliefs, and practices in their context (Denzin & Lincoln, 2018; Rashid et al., 2019). Similarly, in Benner’s (1994) IP, an individual’s experience, views, and lifeworld are both individual and shared, seen as intersubjective, when researchers co-create knowledge with participants (Burns et al., 2022). Additionally, using the IP ontological tenets of embodiment, spatiality, temporality, and care, as previously discussed, enhances a deep exploration, understanding and interpretation of the meaning of human experience to reveal what cannot easily be seen or observed (Benner, 1994).

## Epistemology of FE by Roper and Shapira (2000) and Benner’s (1994) IP

The epistemology or the knowledge creation approach depends on the ontological perspective that guides the research. Roper and Shapira’s (2000) FE and Benner’s (1994) IP embrace social constructivism in knowledge creation. In social constructivism, the researchers seek to understand individuals in the natural settings where they live and work to promote an understanding of people’s historical and cultural backgrounds and how such influence the phenomena of interest (Burns & Peacock, 2019; Denzin & Lincoln, 2018). However, contrary to Benner’s (1994) IP, the researcher in Roper and Shapira’s FE gains a depth of understanding of subjective evidence of a culture, which naturally unfolds as researchers immerse themselves in the research context, the population, linguistic and cultural patterns over a period of time (Roper & Shapira, 2000). Additionally, to capture multiple truths of a phenomenon, diverse views and approaches such as interviews, observations, and examination of cultural artifacts are employed in FE data gathering to promote a thick description (Higginbottom et al., 2013; Roper & Shapira, 2000). During fieldwork, the researcher employs the diffractive way of knowing that allows the researcher to move through a continuum pole of epistemic knowledge, back and forth, to gain a thick description and interpretation of knowledge (Larsen & Schwennesen, 2023).

Similarly, Benner’s (1994) IP emphasizes knowledge creation through several approaches, such as individual and group interviews, dialogues, examination of documents and participant observations. Both Roper and Shapira’s FE and Benner’s IP methodologies acknowledge the researcher as a co-creator of knowledge and an instrument in the research process with the participants (Benner, 1994; Burns et al., 2022; Monrouxe & Ajjawi, 2020; Roper & Shapira, 2000). Therefore, the researcher’s values, beliefs and perceptions are critical in understanding participants’ cultural perspectives and experienced phenomena to create a rich study (Burns et al., 2022; Morse, 2016).

In Roper and Shapira’s (2000) FE and Benner’s (1994) IP research, the body is viewed as an embodiment or space through which sensory feelings and emotions are expressed (Schliewe, 2020). Moreover, in both methodologies, data is gathered and interpreted within the time and place from which data is generated (Benner, 1994; Robinson, 2012). Consequently, the researcher must capture the embodied expressions and the time and place of such experiences to promote a deep interpretation of people’s lifeworld. Based on this imperative, both can achieve theoretical generalization with the transferability of findings to other contexts, promoting the utility of results when a thick or rich description of the phenomena, population and context is achieved (Benner, 1994; Roper & Shapira, 2000).

## Axiological Underpinning of Roper and Shapira’s (2000) FE and Benner’s (1994) IP

Axiology aims to understand the values that guide a research methodology (Curtis & Keeler, 2022b). By employing Roper and Shapira’s (2000) FE methodological approach, one explores a collective cultural experience of a phenomenon to promote a thick description that could advance cultural perspectives in a subculture (Higginbottom et al., 2013; Roper & Shapira, 2000). Such a cultural perspective could enhance acknowledging people’s cultural beliefs and practices to improve the quality of nursing care and health outcomes in a subculture. In contrast, Benner’s (1994) IP could be employed to explore the meaning of an experienced phenomenon to reveal issues taken for granted in everyday life (Frechette et al., 2020). The meanings of such experiences could also be understood and applied in practice to improve nursing care and health outcomes. As both methodologies are co-created by researchers and participants, both are value-bound, and a researcher’s worldview could influence the research outcome. As such, reflexivity becomes a vital practice in both methodologies, enhancing the researcher’s self-awareness and transparency about personal perception and biases that could impact the research process (Burns & Peacock, 2019; Curtis & Keeler, 2022b; Hammersley & Atkinson, 2019; Richards & Morse, 2013).

### Sampling Methods

Both FE by Roper and Shapira (2000) and Benner’s (1994) IP employ the purposive sampling technique to promote the selection of participants who possess in-depth knowledge of the phenomena and who are willing to share their knowledge with the researcher (Benner, 1994; Higginbottom et al., 2013; Roper & Shapira, 2000). The sampling technique in Roper and Shapira (2000) ensures that diverse characteristics of the phenomena under review are represented to enhance heterogeneous and rich data. In Benner’s (1994) IP, a homogenous group is sampled to reveal the meaning of a shared experience. However, unlike in most of Benner’s (1994) IP research, in Roper and Shapira’s (2000) FE, snowballing sampling complements purposive sampling and is employed when participants nominate other individuals who meet the eligibility criteria in sampling (Higginbottom et al., 2013; Roper & Shapira, 2000). The number and characteristics of the participants in the subculture or group determine the sampling method employed in FE (Higginbottom et al., 2013).

Unlike in Benner’s (1994) IP, where the contact is directly between the researcher and the participants, in FE by Roper and Shapira (2000), key informants act as gatekeepers and mediators between the researcher and the potential participants (Higginbottom et al., 2013; Roper & Shapira, 2000). Consequently, key informants ensure the researcher gains access to the research context and facilitate the recruitment of participants who possess experience of the phenomena of interest, especially if the research context is unknown to the researcher (Roper & Shapira, 2000). Therefore, the researcher must be open to key informants on issues related to the research aim, purpose, benefits, harm, and characteristics of potential participants needed for the study. Such fundamental information ensures that appropriate participants with the characteristics a researcher aims for are selected to enhance thick data.

### Sample Size

As with other qualitative research, the sample sizes in both methodologies are usually small compared to quantitative research. The sample size in Roper and Shapira’s (2000) FE is small, although Roper and Shapira (2000) do not specify the exact number. However, data saturation determines sample size, which in most cases is not spontaneous in FE described by Roper and Shapira (2000). In Roper and Shapira’s (2000) FE, insights and knowledge are drawn from initial participant observations and interviews, and used to expand on future interviews. Data gathering is then refined over time to accommodate identified themes until saturation is achieved. Thus, data saturation is achieved when generated themes are supported with substantive data with no new or contradictory information (Cruz & Higginbottom, 2013; Guest et al., 2006).

Similarly, in Benner’s (1994) IP methodology, saturation is influenced by the interpretive process where information retrieved from the data of the first few observations, narratives and interviews allows for more specific and focused questions in later narratives and interviews and more direct observations. The back-and-forth movement from an incomplete to a complete understanding in the interpretive process allows for multiple interviews and observations that promote a deeper insight into the participant’s information and the researcher’s co-creation of the findings (Benner, 1994; Crist & Tanner, 2003). Saturation occurs when the interpretation of data is evident and rich, and themes that have been constructed are supported by data that answer the research questions, with the final analyzed data showing no new themes or meaning identified (Benner, 1994).

### Data Gathering

Both methodologies aim for a large amount of data within a short timeline (Benner, 1994; Roper & Shapira, 2000). In FE by Roper and Shapira (2000), the brief period in the field is compensated by intense data gathering through interviews, observation, field notes, reflective memos, examination of documents (cultural artifacts, epidemiological data, patient records, test results, maps), and audio and video recordings to promote a thick description (Fetterman, 2019; Higginbottom et al., 2013; Roper & Shapira, 2000). Semi-structured interviews allow the researcher to listen, hear, and document cultural behavioral patterns (Rashid et al., 2019). However, participants may not always explain their actions and thoughts during an interview (Andreassen et al., 2020; Rashid et al., 2019). Thus, participant observation provides insight into actual socio-cultural practice within a context and how documented interviews are reflected in people’s actions and behavior (Rashid et al., 2019).

The researcher also employs a selective observation in FE, with observations centered on issues related to the phenomena of interest, requiring a checklist of what is to be observed (Higginbottom et al., 2013; Roper & Shapira, 2000). Due to the short fieldwork, the researcher in FE takes the observer-as-participant stance, encouraging participant observation with limited interaction (Higginbottom et al., 2013; Roper & Shapira, 2000). Therefore, Roper and Shapira (2000) argued that brief contact with the participants reduces the opportunity to validate participants’ observations, limiting a deep understanding and interpretation of a phenomenon. Nevertheless, comparing one’s interpretation of a phenomenon with participants’ views could reduce this limitation and enhance a researcher’s accurate interpretation of a phenomenon (Roper & Shapira, 2000). In Roper and Shapira’s (2000) FE, field notes are employed to record participant observation details, such as date, time, and exact observation, to enhance triangulation, transparency, and richness of the data (Rashid et al., 2019). In addition, the researchers also use reflective memos to document their reflections, thoughts and ideas during data gathering, observation and after interviews, which represent the researcher’s immediate thoughts and interpretations in the research process (Roper & Shapira, 2000).

Contrarily, in Benner’s (1994) IP interviews, individuals share their experiences through a natural storytelling setting, narrating their feelings, situations, occurrences, and everyday life activities. In addition, data could be gathered through group discussions, participants’ observations, and field notes (Benner, 1994; Benner et al., 1996; Burns & Peacock, 2019; Dos Santos et al., 2016). Multiple data gathering and triangulation approaches are crucial and encouraged as each data gathered informs subsequent data and influences the interpretive process, enhancing the interpretation of an experienced phenomenon (Benner, 1994). Benner (1994) emphasizes the need for multiple interviews that allow for an in-depth unveiling of the phenomena under study, which also prevents emphasis on non-recurrent statements and themes. Additional researchers are also required in Benner’s (1994) IP to enhance rigorous and accurate interpretation of the meaning of participants’ experiences, given the high volume of data.

In interviews conducted using both methodologies, emphasis is placed on the use of the six senses of sight, hearing, taste, touch, smell, and emotion, which allows researchers to pay attention to non-verbal cues that foster understanding of their mood and that of the participant while enhancing understanding of peoples’ experience of a phenomenon (Benner, 1994; Benner et al., 1996; Crist & Tanner, 2003; Cruz & Higginbottom, 2013; Frechette et al., 2020; Sharma et al., 2009). Additionally, the researcher is encouraged to listen and use silence in some instances to allow for the unveiling of the phenomena under review (Benner, 1994; Dos Santos et al., 2016; Rashid et al., 2019).

### Data Analysis

Data analysis in both methodologies begins while data is being gathered and iteratively continues until a rich interpretation of data is achieved (Benner, 1994; Robinson, 2012; Roper & Shapira, 2000). In FE by Roper and Shapira (2000) and Benner’s (1994) IP, there is a verbatim transcription and analysis of the interview, observations, field notes, and reflective memos, which add to the richness of the data. Additionally, in FE by Roper and Shapira (2000), gathered documents such as cultural artifacts, epidemiological data, patient records, test results, and maps are also integrated into data analysis to make sense of the cultural norms being explored.

Data analysis in FE, according to Roper and Shapira (2000), follows five steps, namely, (a) coding for descriptive labels, (b) sorting for patterns, (c) identification of outliers or negative cases, (d) generalizing with constructs and theories, and (e) memoing and reflective remarks. While these steps exist, data analysis in FE by Roper and Shapira (2000) is not chronological but fluid, which allows researchers to move back and forth between the steps of the analysis to enhance a thick description of the phenomenon being studied.

In the first step of coding for descriptive labels, all transcripts, field notes, reflective memos and gathered clinical documents and artifacts are read word by word by the researcher team and coded into meaningful categories to reflect the raw data (Roper & Shapira, 2000). These codes are then compared and contrasted to identify the patterns running through them (Roper & Shapira, 2000). The first level of coding is also applied to reduce the many pages of the data into a manageable size (Roper & Shapira, 2000). The researcher team usually debriefs continually to understand identified new themes in observation and early interviews, which are incorporated in later interviews to enhance the thickness of the data. In the second step of sorting for patterns, the researcher groups the descriptive labels into smaller sets to develop themes while looking for connections and information that ran through the data (Roper & Shapira, 2000). In the third step of identification of outliers or negative cases, the researcher aims to identify outliers or data unrelated to the study, which could play a vital role in the study’s outcomes (Roper & Shapira, 2000). Such outliers are usually not discarded but are preserved until the researcher understands their impact on the research (Roper & Shapira, 2000). In the fourth step of generalizing with constructs and theories, the researcher relates findings to theories and existing literature (Roper & Shapira, 2000). In the fifth step of memoing and remarks, the researcher draws ideas, insight and clarifications from written memos that the researcher documents throughout the research process to ensure transparency in data analysis (Roper & Shapira, 2000).

Data analysis in Benner (1994) continues with verbatim transcription and comparison of the tapes with the transcript to enhance the validity of the transcript. Benner's (1994) approach to data analysis follows five stages, which are elaborated by Crist and Tanner (2003), namely, (1) early focus and lines of inquiry, (2) central concerns, exemplars and paradigm cases, (3) shared meanings, (4) final interpretations, and (5) dissemination of the interpretation. In the first stage of early focus and lines of inquiry, the researchers follow an interpretive process to discuss the transcripts of the first few participants while critiquing and evaluating the interviews and observations evident in the participants’ narratives (Benner, 1994; Crist & Tanner, 2003). During this time, missing and unclear sections in the narrative, interviews and observations are noted for further exploration as researchers remain open to new research questions (Benner et al., 1996; Crist & Tanner, 2003). The initial interpretation of the transcripts by the team guides further interviews and sampling to enhance a deeper understanding of the phenomenon being studied (Benner, 1994; Crist & Tanner, 2003).

In the second stage of central concerns, exemplars and paradigm cases, the research team identifies areas that are of central concern in the transcript based on the team’s initial interpretation and subsequent transcript interviews and observations (Benner, 1994; Crist & Tanner, 2003). The research team first identifies a paradigm case by reading the transcripts holistically and selecting specific transcript components for further analysis and interpretation (Benner, 1994; Crist & Tanner, 2003). Following this, other paradigm cases are developed, analyzed independently, and later compared with the first paradigm case to understand the similarities and differences (Benner, 1994; Crist & Tanner, 2003). This comparison allows the researcher to deeply understand the participants’ way of being in relation to the phenomenon under study (Benner, 1994; Crist & Tanner, 2003). The development of paradigm cases is followed by a thematic analysis, which follows an interpretive process where the researchers move back and forth from understanding a part of a text to understanding the whole text to increase the depth of meaning and interpretation of the text (Benner, 1994; Crist & Tanner, 2003). Subsequently, exemplars, which are relevant verbatim stories or patterns in the narratives with the same or divergent meanings across many participants, allowing for a deep understanding of participants’ experiences, are extracted during the analysis (Benner, 1994). The research team maintains interpretive summaries of writing, revising, and refining their interpretation, which could allow some codes to change with time (Benner, 1994; Crist & Tanner, 2003).

In the third stage of shared meanings, participants’ central concerns become more apparent as the research team begins to observe shared meanings evolving (Benner, 1994; Crist & Tanner, 2003). The team also observe shared meanings within and across participants’ transcripts in the documented interpretive summaries (Benner, 1994; Crist & Tanner, 2003). In the fourth stage of final interpretations, the research team will continue to document interpretive summaries, which will help to explain constructed interpretations and final interviews (Benner, 1994; Crist & Tanner, 2003). These interpretive summaries also allow for a rich interpretation of excerpts and central concern summaries (Benner, 1994; Crist & Tanner, 2003). In the fifth stage of dissemination of the interpretation, the manuscript continues to be refined iteratively through participants’ narratives, field notes of observations and contributions from the research team (Benner, 1994; Crist & Tanner, 2003). A rigorous approach to study interpretation in data analysis can “broaden possibilities for nursing interventions, policymaking, philosophy, and research” (Crist & Tanner, 2003, p. 205).

A study conducted by Strandås et al. (2019b) reflects an appropriate application of Roper and Shapira’s (2000) FE methodology in research, as the authors showed consistency with the methodological underpinning. For example, Strandås et al. (2019b) aimed to gain an in-depth “understanding of nurse–patient relationships by exploring meanings and values behind the beliefs and practices of nurses and patients in Norwegian public home care” (p. 402). The purposive sampling method was used to select 18 participants for interviews. Consistent with FE by Roper and Shapira (2000), the authors used nurse managers as gatekeepers who assisted researchers in accessing facilities and recruitment. Apart from interviews, participant observation and field notes assisted in enhancing the triangulation and trustworthiness of the study, which is consistent with Roper and Shapira's (2000) FE. Findings from the study provide important insight that could be used to enhance therapeutic nurse-patient relationships and high-quality nursing care. Although Roper and Shapira’s (2000) FE methodology can be applied within a relatively short duration of time, Strandås et al.’s (2019b) study was conducted over 8 months. While somewhat longer than usually expected in FE, a likely factor was that the fieldwork extended into six home care facilities.

In the same way, Oerther’s (2023) study that aimed to understand parents’ everyday concerns in caring for their children, intended to uncover barriers to pre-teen flourishing and identify ways to support pre-teen flourishing, was found consistent with Benner’s (1994) IP methodology. Consistent with Benner (1994), the study was conducted in a natural environment using a purposive sampling technique among participants who have experienced the phenomenon of interest, enhancing the richness of the data. Though data gathering was only carried out by one researcher, which is inconsistent with Benner’s (1994) IP methodology, the researcher ensured that probing questions were used to enhance a deep understanding of participants’ experiences. Additionally, participants were interviewed more than once to enhance a holistic understanding and interpretation of their experiences, consistent with Benner’s (1994) IP. The researcher ensured that embodied emotions and non-verbal cues were captured in field notes to promote a deeper interpretation of participants’ experiences. Data analysis was carried out by a team of researchers using Benner’s (1994) IP approach, elaborated by Crist and Tanner (2003). Findings from this study provide valuable insight into approaches to support parents and develop interventions and policies that could promote pre-teen thriving outcomes.

### Trustworthiness

The approach to trustworthiness is similar in both FE and IP, recognizing that researchers must first acknowledge their research stance and the ontological and epistemological philosophy that shapes their research (McGinley et al., 2021). Such statements allow researchers to establish their worldview and position in knowledge creation, strengthening the research methodology (Jackson, 2013). Additionally, both methodologies follow the criteria of credibility, transferability, dependability, and confirmability outlined by Lincoln and Guba (1985).

To meet the criteria of credibility, which ensures that the findings are congruent with the participant’s experience, the researcher in both methodologies must have the academic, theoretical and experiential skills to conduct FE or IP research (Forero et al., 2018). Additionally, researchers in both Roper and Shapira’s (2000) FE and Benner’s (1994) IP are to have a prolonged engagement within the research context through observation and immersion in the data analysis to promote a multidimensional understanding of the data. Pilot testing of the interview questions is also done to enhance the validity of the research instruments in both methodologies. Constant debriefing in both FE by Roper and Shapira (2000) and IP by Benner (1994) of the research methodology, methods, transcripts, and findings with colleagues also helps ensure trustworthiness. Member checking of transcripts with participants is performed in both methodologies to validate the transcripts, which is also crucial to validating researchers’ interpretation and enhancing transparency (Benner, 1994; Roper & Shapira, 2000).

To meet the transferability criteria, which ensures that findings could apply to other contexts, researchers must incorporate a purposive sampling technique, which ensures participants are selected based on their experience with the phenomenon of interest to enhance rich data (Benner, 1994; Roper & Shapira, 2000). The researcher also ensures that data saturation is achieved when additional findings do not generate new information or insight into the research question (Benner, 1994; Roper & Shapira, 2000). The dependability criteria ensure that participants’ data support findings and interpretations of data are met when there is a detailed description of the study protocol, the data gathering approach and accuracy in the data coding (Forero et al., 2018). The confirmability criteria ensure that the meaning of the research findings is not changed because of the researcher’s knowledge, views, and experience, given that researchers in FE and IP are co-creators of knowledge with participants (Lincoln & Guba, 1985). This criterion is met by using several triangulation and reflective memos, ensuring that the researcher’s opinion and bias do not influence the study process (Benner, 1994; Roper & Shapira, 2000). An audit trail that allows for detailed documentation of the research process, such as the data gathering process, data analysis, and interpretation, also ensures confirmability (Forero et al., 2018).

Given the co-creative nature of knowledge in both FE by Roper and Shapira (2000) and Benner’s (1994) IP methodology, reflexivity is an essential element of confirmability that allows the researcher to self-consciously reflect on how the researcher’s beliefs, values and judgement influence the research process, analysis and findings, given that knowledge is co-created with the researcher. Reflexivity also allows the researcher to be transparent on how the researcher, the methodology and data gathering methods influenced the research process and findings (Benner, 1994; Roper & Shapira, 2000). A researcher brings personal values, ideas and beliefs into the research, which cannot be easily detached from the researcher (Cruz & Higginbottom, 2013). A transparent acknowledgment of these personal values is vital in Roper and Shapira’s (2000) FE, especially if the researcher is familiar with or shares the same culture being studied. Thus, reflexivity enhances the validity of the data and shows that the findings reflect the participants’ views, not that of the researcher (Benner, 1994; Roper & Shapira, 2000).

## Justification for the Use of Roper and Shapira’s (2000) FE and Benner’s (1994) IP

Choosing between these two methodologies in nursing research depends on the research question guiding the phenomenon of interest, the research problem, and the researcher’s worldview. Using FE described by Roper and Shapira (2000) or Benner’s (1994) IP appropriately in research allows for a detailed interpretation and understanding of the phenomena of interest and resonance with individuals or groups who share such experiences. For example, FE is an evolving methodology which has been employed by several nurse researchers to explore critical issues in nursing practice (Strandås et al., 2019a; Sørstrøm et al., 2023). In FE, a researcher’s familiarity and prior theoretical knowledge are vital to enhancing an in-depth understanding of the phenomena under review (Higginbottom et al., 2013; Roper & Shapira, 2000). Such knowledge allows a researcher to formulate appropriate research questions that clearly outline the study population’s needs and the nature of the investigation. Moreover, prior knowledge of the research context and a shared language could promote a researcher’s ease of access, fostering trust in the study setting (Wall, 2015). However, the challenge of a shared value and belief could promote a non-acknowledgment of the cultural patterns and principles within the context. Thus, one’s understanding of her position as a researcher encourages transparency and a reduction of this limitation (Andreassen et al., 2020).

In addition, the methodological underpinning of Roper and Shapira (2000) FE provides diverse methods to understand and explore multiple realities in a specific cultural context. For example, the combination of participant observation, field notes, interviews, and audiovisual recording promotes a thick description of the cultural norms, the context, the participants, and the phenomena of interest. Such diverse methods in research would allow for a thick and in-depth exploration and understanding of the complex and multilayered nature of groups’ cultural beliefs and practices. Through this in-depth exploration, cultural issues taken for granted could be revealed, and insights are drawn on approaches to enhance culturally appropriate care and nursing practice to promote health outcomes. Similarly, diverse methods promote triangulation, transparency, and the rigor of research findings.

On the contrary, Benner’s (1994) IP is well suited for exploring individuals’ experiences of health and illness, especially if such a phenomenon is poorly researched or understood (Burns & Peacock, 2019; Polit & Beck, 2021). Benner’s (1994) IP is also well suited to explore and provide a rich understanding, unveiling, and interpretation of issues that are taken for granted in nursing practice, even when such practices appear to function smoothly and transparently (Benner, 1994; Dos Santos et al., 2016). In using Benner’s (1994) IP, the philosophical tenets of IP, which include embodiment, spatiality, temporality, and care, could promote a deeper understanding of the meaning of an individual’s experience of a phenomenon from the individual’s perspective, which also could promote nursing practice (Burns et al., 2022; Dos Santos et al., 2016). Benner's (1994) IP has been used by several nurse researchers to understand people’s everyday experience of a phenomenon (Isaak & Paterson, 1996; Leung et al., 2012; Massimo et al., 2013).

## Conclusion

Selecting an appropriate qualitative methodology in nursing research could be challenging for novice nurse researchers who may lack the background and in-depth knowledge to apply such methodologies in research. In this paper, we have discussed the historical, ontological, epistemological, axiological, and methodological underpinnings of FE described by Roper and Shapira (2000) and IP described by Benner (1994). We have also outlined the similarities and differences between both methodologies while justifying using Roper and Shapira’s (2000) FE and IP described by Benner (1994) in research. While both Roper and Shapira (2000) and IP described by Benner (1994) are qualitative approaches that embrace the interpretation of meanings in human experiences, Roper and Shapira (2000) explore the collective cultural experiences of a group while Benner’s (1994) IP focuses on providing an in-depth understanding and interpretation of an experienced phenomenon. Moreover, while the methodological underpinnings of Roper and Shapira (2000) and IP described by Benner (1994) are similar to some extent, there are some differences in their application in research. For example, both methodologies emphasize the use of diverse methods in data gathering, such as interviews, participant observations and field notes. However, the approach to which these methods are applied in research using these methodologies differs. Therefore, this paper has simplified and clarified the application of these methodologies in research for novice researchers. Appropriate application of these methodologies in research could enhance a rich exploration and interpretation of the human experience of phenomena that could promote health outcomes and nursing practice.
